# Orexin and Lifestyle Habits: A Meaningful Connection Among Nutrition, Physical Activity, and Sleep Pattern in Health and Diseases

**DOI:** 10.3390/ijms26188980

**Published:** 2025-09-15

**Authors:** Ersilia Nigro, Francesca Argentino, Giuseppe Musumeci, Aurora Daniele

**Affiliations:** 1 Department of Pharmaceutical, Biological, Environmental Sciences and Technologies, University of Campania “Luigi Vanvitelli”, Via G. Vivaldi 42, 81100 Caserta, Italy; ersilia.nigro@unicampania.it; 2 CEINGE-Biotechnologies Advances S.c.a r.l., Via G. Salvatore 486, 80145 Naples, Italy; argentino@ceinge.unina.it (F.A.); aurora.daniele@unina.it (A.D.); 3 Center for Neuroscience, International School of Advanced Studies, University of Camerino, 62032 Camerino, Italy; 4 Department of Biomedical and Biotechnological Sciences, Anatomy, Histology and Movement Sciences Section, School of Medicine, University of Catania, Via S. Sofia 87, 95123 Catania, Italy; 5 Research Center on Motor Activities (CRAM), University of Catania, 95123 Catania, Italy; 6 Department of Molecular and Biotechnological Medicine, University of Naples “Federico II”, 80131 Naples, Italy

**Keywords:** orexin, physical activity, sleep, nutrition, neurodegeneration

## Abstract

Orexin is a neuropeptide produced in the hypothalamus that plays a key role in regulating slee—wake cycles, energy metabolism, feeding behavior, and physical activity. It exists in two forms, orexin-A and orexin-B, which bind to G protein-coupled receptors OX_1_R and OX_2_R with differing affinities. Orexin signaling is widespread in the brain and extends to peripheral tissues, including adipose tissue. Its involvement in hypothalamic and extrahypothalamic circuits suggests a broad role in homeostatic regulation. Dysfunctions in the orexinergic system are implicated in neurodegenerative diseases such as Alzheimer’s, Parkinson’s, and multiple sclerosis, particularly through mechanisms involving sleep disturbances and neuroinflammation. This study examines how orexin influences neural circuits related to arousal, motivation, and motor control. It also explores how physical activity stimulates orexin release, enhancing neuroplasticity and cognitive resilience. In addition, orexin’s role in reward-related feeding, genetic susceptibility to obesity, and brown adipose tissue thermogenesis is discussed. Overall, the orexinergic system represents a vital neurochemical link between physical activity, metabolism, and cognitive health. Although many of its mechanisms remain to be clarified, its central role in integrating energy balance and behavioral responses makes it a promising target for future therapeutic strategies.

## 1. Introduction

An active lifestyle promotes both physical and mental health due to the release of several soluble factors, which in turn regulate the production of both additional peripheral and central proteins, such as orexin. Orexin, also known as hypocretin, is a neuropeptide produced in the lateral hypothalamus that is crucial for regulating wakefulness, appetite, and energy expenditure [1]. Furthermore, an important role of orexin has been identified in other behaviors, such as the promotion of arousal and drug seeking [2,3,4,5]. It exists in two forms, orexin-A and orexin-B, which interact with G-protein-coupled receptors OX_1_R and OX_2_R. These receptors are widely distributed throughout the brain, influencing various physiological processes [6], i.e., the regulation of arousal and energy homeostasis, in several brain regions including the hypothalamus, locus coeruleus, and dorsal raphe nucleus [7]. Moreover, orexin signaling expands to several peripheral tissues, including adipose tissue [8] where both receptors are expressed. The two forms of the neuropeptide present different affinities to their two receptors and this aspect has garnered attention for its possible implication in the wide range of behavioral effects of orexin. In detail, the orexin system is implicated in several physiological functions including energy homeostasis (reviewed in [9]), nutrition (reviewed in [10,11]), arousal or wakefulness [12] (reviewed in [13]), reward (reviewed in [14]), stress and anxiety (reviewed in [15]), and reproduction (reviewed in [16]). Furthermore, it has long been known that orexinergic signaling regulates the hypothalamic–pituitary–adrenal (HPA) axis (reviewed in [16]) and that its activity in the female hypothalamus is influenced by specific moments in the reproductive cycle [17,18,19,20]. Among the wide variety of effects exerted by orexin, its regulation of energy balance is linked to physical activity (PA), particularly to spontaneous physical activity (SPA) and non-exercise activity thermogenesis (NEAT) [21,22]. The World Health Organization (WHO) defines PA as any bodily movement produced by skeletal muscles that necessitates energy expenditure [23]. This definition comprises all forms of movement of light-, moderate-, and vigorous-intensity, all recognized for their health benefits. The concept of NEAT encompasses all forms of energy expenditure that are not directly associated with structured exercise. This includes activities such as standing and fidgeting, which are often overlooked in the context of energy expenditure and PA [24]. Complementarily, SPA includes any type of PA that does not fall within the category of voluntary exercise and that results in NEAT [25].

PA is well-documented for its beneficial impact on brain health, with positive implications on various cognitive and physiological processes, such as memory, attention, and executive functions [6,26]. This is largely attributed to the promotion of neuroplasticity [27] which pass through the regulation of secretion of several mediators. For example, exercise stimulates the production of neurotrophic factors such as brain-derived neurotrophic factor (BDNF) and insulin-like growth factor 1 (IGF-1), which support neuronal growth, survival, and synaptic plasticity [28,29]. Additionally, PA increases cerebral blood flow [30], providing the brain with more oxygen and nutrients, and helps to reduce inflammation and oxidative stress, both of which are implicated in cognitive decline and neurodegenerative diseases [29].

Orexin plays a critical role in these processes being proposed as one of the molecular links between PA and neurodegeneration. The interaction between PA and orexin is particularly noteworthy, as exercise has been shown to stimulate orexin production [31], which in turn promotes further PA and cognitive benefits. Overall, the integration of regular PA into daily routines, supported by orexin signaling, is a powerful strategy for maintaining and improving brain health across the lifespan. Given orexin’s central role in maintaining arousal and energy balance, it is important to thoroughly investigate its function in relation to PA. This study aims to explore the bidirectional mechanisms linking orexin to PA and nutrition, as well as the variations in orexin levels among different physiological and pathological states, and the potential therapeutic implications of these findings.

## 2. Brief Overview on Orexins: Structure and Functions

**Figure 1:**
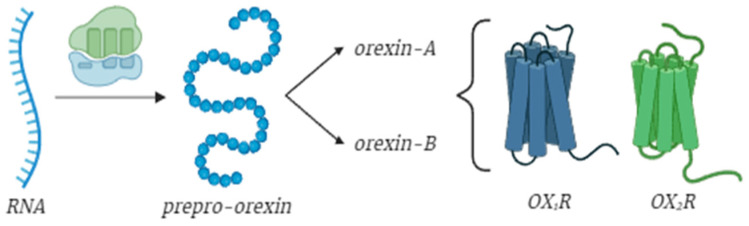
Orexin protein and orexin receptors. Orexin-A and orexin-B are generated from the cleaving of prepro-orexin and interaction with their two receptors, OX_1_R and OX_2_R. Image created in BioRender.com.

Orexins are encoded as a single polypeptide precursor named “prepro-orexin”, which is then cleaved into two neuropeptides: orexin-A and -B [32]. Orexin-A is a 33-amino-acid peptide characterized by a N-terminal pyroglutamyl residue, two intrachain disulfide bonds, and C-terminal amidation (Figure 1). Remarkably, this structure is fully conserved across mammalian species, including humans, gorillas, rats, mice, cows, pigs, sheep, dogs, seals, and dolphins [32]. Similarly, orexin-B, a 28-amino-acid linear peptide with C-terminal amidation, also exhibits a highly conserved structure among mammals. While the C-terminal half of orexin-B closely resembles that of orexin-A, the N-terminal half shows greater variability [33] (Figure 1). As previously stated, orexin exerts its effects by binding to two types of receptors, OX_1_R and OX_2_R, which are both G-protein-coupled receptors and are present in multiple regions of the brain and throughout the entire organism, with a differential expression [34,35]. Phylogenetically, OX_1_R emerged later than the other, suggesting its involvement in more complex mechanisms [32]. Their activation is fundamental to all of orexin’s diverse functions. For instance, the orexinergic projections in the locus coeruleus play a critical role in promoting arousal and wakefulness [13]. Projections to the dorsal raphe nucleus contribute to the regulation of mood, anxiety, and sleep [21,36], while connections to the tuberomammillary nucleus are essential for maintaining wakefulness [13]. Additionally, orexinergic neurons inhibit sleep-promoting neurons in the ventrolateral preoptic area, thus regulating the sleep–wake cycle [37]. Further projections to the paraventricular nucleus are involved in the regulation of autonomic functions and energy homeostasis [8]. The ventral tegmental area and nucleus accumbens receive orexinergic inputs that are integral to the modulation of reward, motivation, and feeding behaviors [38]. In higher cognitive processes, orexinergic neurons project to the prefrontal cortex, influencing decision-making and executive functions [39]. Emotional regulation is mediated through projections to the amygdala [40]. Lastly, orexinergic projections to the spinal cord modulate pain perception and autonomic functions [41], highlighting the extensive reach and influence of the orexinergic system throughout the central nervous system. Considering the wide-reaching effects of orexin across these diverse regions, it is clear how critical it is to account for the varied roles orexin may play in multiple aspects of life, from cognitive function to metabolic regulation.

## 3. Molecular Mechanisms and Effects of Orexin Receptors

**Figure 2:**
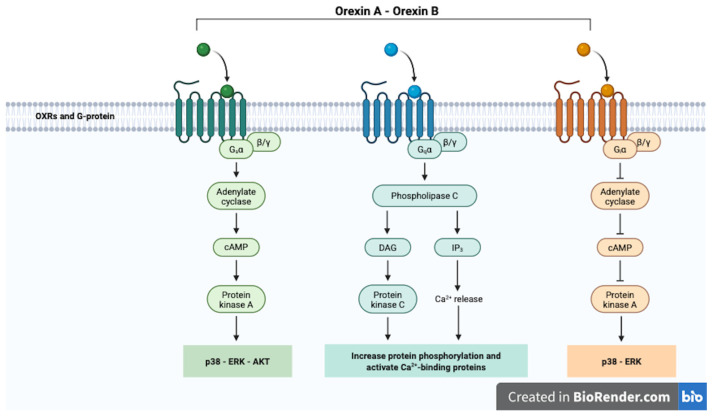
Orexin receptors signaling pathways. Image created in BioRender.com.

Orexin exerts its effects primarily by binding to and activating two G-protein coupled receptors, OX_1_R and OX_2_R, proteins of 425 and 444 aa, with 64% amino-acid identity. These receptors, when activated, initiate intracellular signaling cascades that regulate various physiological processes, including sleep–wake cycles, feeding behavior, reward, and energy balance. Orexin-A and orexin-B, the two orexin peptides, bind to OX_1_R and OX_2_R with varying affinities. Orexin-A binds to both receptors with high affinity, while orexin-B primarily binds to OX_2_R with high affinity [42]. More recently, the dimerization in homo- or heterodimers of OXRs has been demonstrated, with the suggestion that it might regulate some specific responses [43]. In vitro studies have provided detailed insights into the molecular pathways through which orexin regulates energy balance and neuronal activity. After the binding to OXRs, orexins generate subsequent activation of G-protein subtypes (Figure 2). Initial studies identified G_q_ coupled with PLC-mediated calcium elevation [33,44,45] in the regulation of feeding behavior. The G_q_-mediated PLC/PKC cascade ultimately leads to phosphorylation-based activation of the ERK pathway [46]. Indeed, studies have demonstrated that orexin treatment significantly increases the intracellular Ca^2+^ concentration ([Ca^2+^]_i_) in cells overexpressing OX_1_R and OX_2_R, an effect that is mainly triggered by activation of the classical phospholipase C (PLC) cascade (PLC-IP3/DAG) [47,48,49].

Subsequently, more signaling pathways have been identified. Indeed, orexins can also activate G_s_ and G_i_ proteins: G_s_/AC-mediated PKA cascade and JNK, AKT or p38 activation via G_i_-proteins [50,51,52].

Additionally, orexin signaling can involve the phospholipase D (PLD) and mitogen-activated protein kinase (MAPK) pathways, which contribute to diverse cellular responses such as neurotransmitter release, cellular growth, and stress adaptation [52]. Ultimately, the orexinergic signaling also comprises of the receptor association with GPCR kinases such as GRK2 and GRK5, and recruitment of the β-arrestin proteins, which act as signaling scaffolds [53,54,55].

The localization of the receptors suggests the multifaced roles of orexins primarily but not only in the brain. Notably, the two receptors are widely distributed throughout the brain (hypothalamus, brainstem, cerebral cortex, basal ganglia, hippocampal formation, thalamus) and less present in non-brain areas like the retina, gastrointestinal tract, adipose tissue, or reproductive organs. Researchers are studying orexins’ regulation and mechanism of action in multiple diseases, from neurodegenerative to metabolic and immune/inflammatory conditions. For example, orexins can regulate proliferation, apoptosis, and autophagy in ischemia–reperfusion injury through ERK1/2 and MAPK/ERK/mTOR pathways [56]. The antitumor effect of orexin was first reported in 2004 by Rouet-Benzineb et al. [57] that determined the inhibitory property of orexins associated with the induction of mitochondrial apoptosis. Again, the molecular pathways involved are MAPK/ERK/mTOR. An additional key molecular mechanism at the basis of the beneficial influence of orexin on the brain is its ability to induce BDNF expression. BDNF is one of the most studied neurotrophic factors of the nervous system and it exerts its functions by binding the TrkB receptor [58,59]. It is involved in adult neurogenesis and nerve maturation, as well as in the regulation of synaptic transmission and plasticity and memory and learning [58,60,61]. It was shown that the microinjection of orexin into the rostral ventral medulla induces an increase in the expression of BDNF [62]. The numerous benefits of this factor are not strictly limited to physiological conditions. In fact, in the context of Alzheimer’s disease (AD), research is focusing on BDNF as a potential treatment and protective factor against neurodegeneration. Several studies reported improvements both at the microscopic (i.e., the pathological protein accumulation) and macroscopic (i.e., the cognitive performances) level in Alzheimer’s conditions when BDNF levels were higher [63,64,65]. The synthesis of BDNF induced by orexin may have therapeutic implications also in another neurodegenerative condition: Parkinson’s disease (PD). This disease is characterized by both cognitive and motor deficits resulting from the loss or degeneration of the dopaminergic neurons of the substantia nigra in the midbrain and from the formation of abnormal protein deposits known as “Lewy bodies” [66]. BDNF, that is also reduced in Parkinson’s condition, may be useful for preventing the pathological loss and providing neuroprotection [67]. This indirect link among orexin secretion, BDNF production and neurodegeneration makes orexin as a relevant target to be addressed in the management of disorders such as Parkinson’s and Alzheimer’s diseases; we will discuss this point further.

On the other hand, it would be useful to further investigate the interactions between progesterone and orexin in women and understand whether there is a distinction between the two receptors OX_1_R and OX_2_R in relation to functions such as depressive behavior and cognitive functions. In women, during the menstrual cycle, there is a fluctuation in circulating levels of estradiol, progesterone, and the production and receptor activity of the peptide orexin. Indeed, depending on the phase of the estrous cycle, the transcription of prepro-orexin mRNA, post-translational modifications of the orexin peptide, and the abundance of orexin receptors vary specifically for each brain region [68]. The most significant changes occur in the hypothalamus, considered the starting point of the hypothalamic–pituitary–gonadal axis and the hub of orexin-producing neurons.

## 4. Orexin Increases Physical Activity Levels

As above-mentioned, orexins stimulate both feeding and energy expenditure. The latter effect is mainly induced by increasing spontaneous physical activity. Some studies explored the effects of orexin administration on NEAT: when injected into the hypothalamic paraventricular nucleus (PVN), the neurotransmitter significantly increases NEAT, by enhancing SPA [69,70]. Additional studies have analyzed the effects of orexin administration in various brain regions, including the locus coeruleus, ventricles, nucleus accumbens, and substantia nigra [71,72,73,74]. The results of these studies consistently indicate an increase in SPA, which is directly correlated with NEAT. In another study, Novak and colleagues analyzed the differences in the propensity to remain lean between high- and low-capacity running rats, focusing on energy expenditure and NEAT [75]. The authors also tried to find the underlying mechanisms of the differential sensitivity to the exercise-activating properties of the rats by examining the orexinergic system. They found no differences in the expression levels of orexin or in the mRNA levels of its receptors, suggesting that there may be an increase in orexin degradation in low-capacity running rats.

The most plausible mechanism by which orexins promote locomotor activity is through the activation of pathways that enhance arousal and motivation [1]. The neurons most involved and essential in promoting arousal and maintaining wakefulness by orexins are located in the lateral hypothalamus. Here, orexins also activate a subset of lateral hypothalamic neurons that are essential for movement [76] and interact with several neurotransmitter systems, including norepinephrine, dopamine, and serotonin, which are all involved in regulating mood and motivation [21]. Interestingly, orexin production is influenced by circadian rhythm: orexin neurons are in contact with the suprachiasmatic nucleus [77]. The increase in orexin during exercise depends on the time of day and predisposes to wake-related increases in both glucose metabolism and the cardiovascular system [78]. Furthermore, orexin-A levels have been shown to increase in rodent cerebrospinal fluid (CSF) and human plasma [31,79] in response to exercise. Conversely, blockade of orexin receptors reduces core body temperature after a single 20 min exercise session [80]. Furthermore, Koumar O.C. et al., 2023 have shown that both in rats, as well as in humans, the core body temperature varies according to the time of exercise without orexin-A production changes [81].

### 4.1. Orexin and Motor Control

The widespread and dense innervation by orexinergic fibers suggests a robust modulatory influence on motor circuits, contributing significantly to the coordination and execution of motor behaviors [82]. In the motor cortex, orexin receptors exhibit a distinct distribution pattern, with OX_1_R receptors predominately localized in cortical layers III and IV, while OX_2_R receptors are largely found in layers V and VI [83,84]. This differential receptor expression potentially underpins complex modulation of motor cortical outputs involved in voluntary movement initiation and execution [82]. Furthermore, within the spinal cord, orexinergic fibers notably innervate the ventral horn, directly influencing motor neurons responsible for the execution of muscle movements. This spinal modulation involves excitatory interactions primarily mediated through the activation of OX_1_R receptors, thereby facilitating increased motor neuron excitability and enhancing motor activity [85,86].

The basal ganglia, a critical network implicated in the regulation of motor control and the integration of motor information, also receives substantial orexinergic innervation. Specifically, orexin-A and orexin-B have been shown to increase neuronal firing rates in the subthalamic nucleus, a pivotal nucleus within the basal ganglia circuitry [87]. This excitatory modulation via OX_1_R and OX_2_R receptors suggests that orexinergic signaling significantly influences basal ganglia-mediated motor control processes such as movement initiation, modulation of muscle tone, and coordination of complex motor sequences [88,89]. Moreover, orexin exerts pronounced effects within the lateral vestibular nucleus, an essential brainstem region involved in the maintenance of postural balance and coordination. Orexin-A directly excites lateral vestibular nucleus neurons through mechanisms involving Na^+^-Ca^2+^ exchangers and inward rectifier K^+^ channels, thus enhancing the neuronal responsiveness crucial for vestibular reflexes and motor equilibrium [90]. These actions are particularly important during motor tasks demanding high postural and balance control, highlighting orexin’s role in motor adaptation to challenging environmental contexts [91]. The functional significance of orexinergic modulation in motor control is underscored by pathological states arising from orexin deficiency, such as narcolepsy–cataplexy, characterized by sudden and transient loss of muscle tone [92]. This relationship highlights orexin’s pivotal role in stabilizing motor function and maintaining posture during both static and dynamic states. Experimental evidence further supports the idea that orexin contributes significantly to motor behaviors by regulating the excitability and synchronization of motor neurons at multiple neural levels, thus playing a vital role in motor planning, initiation, and execution [93,94].

Overall, the extensive innervation and receptor distribution, combined with robust electrophysiological and behavioral evidence, firmly establish orexin as a critical modulator of motor functions, representing a fundamental neurochemical pathway involved in orchestrating motor behaviors, providing a potential therapeutic target for addressing motor deficits associated with various neurological disorders [3,95].

### 4.2. Correlation Between Orexin, Physical Activity, and Neurodegenerative Disorders

Orexin’s role in diseases has garnered increasing interest, also for its interaction with PA especially in neurodegenerative disorders [29]. Physical exercise is well-documented for its neuroprotective effects, which include the enhancement of neuroplasticity, reduction of neuroinflammation, and promotion of neurogenesis [27,96]. These benefits are crucial for preventing and managing neurodegenerative conditions such as Alzheimer’s disease, Parkinson’s disease, and multiple sclerosis (MS). The interaction between PA and orexin plays a significant role in these neuroprotective effects.

Regular PA has been associated with a reduced risk of AD [97] with protective effects by promoting better sleep patterns, reducing amyloid-β (Aβ) accumulation, and enhancing synaptic plasticity [98,99]. However, the relationship between orexin levels and AD is controversial, with studies finding higher orexin levels in patients [100], lower orexin levels in patients [101], or even no differences between patients and healthy controls [102,103]. Although the neurotransmitter appears to be physiologically linked to better cognitive outcomes and neuroplasticity, in the Alzheimer’s pathological scenario it may be associated with impaired cognitive function and increased Aβ accumulation [104,105]. This dualistic role of orexin highlights the importance of further studies to better comprehend the involvement of the orexinergic system in pathological mechanisms, considering the importance of physical exercise in these processes.

In PD, PA has been shown to improve motor function, balance, and quality of life [106]. Orexin contributes to these benefits by enhancing dopaminergic signaling [107] and neuroplasticity [108]. Exercise also increases the expression of neurotrophic factors such as BDNF, which supports neuronal survival and function [109]. The interplay between orexin and these neurotrophic factors may be a key mechanism through which exercise exerts its neuroprotective effects in PD, where orexin deficiency is linked to non-motor symptoms like fatigue and sleep disturbances [110,111].

In MS, studies have shown that physical exercise can improve the quality of life and the symptoms affecting patients [112]. Regarding orexin levels, they can be both elevated and reduced depending on the disease state and specific brain regions affected [113]. Research indicates that orexin levels in MS patients may vary, with some studies reporting lower cerebrospinal fluid orexin-A levels in MS patients with hypothalamic lesions, which are associated with hypersomnia and fatigue [114]. Conversely, other studies have found increased orexin-A levels in the serum of MS patients [115], suggesting a potential compensatory mechanism to counteract disease symptoms. The molecular mechanisms behind the intricate relationship between orexin and PA and neurological benefits are well-documented [7]. A well-established mechanism is the stimulated release of orexin by exercise, which in turn promotes the production of BDNF, IGF-1, and other neurotrophic factors. These molecules enhance synaptic plasticity, neurogenesis, and mitochondrial function, contributing to improved cognitive function and resilience against neurodegenerative processes.

## 5. Orexin and Nutrition

The precise role of orexins in food intake remains to be fully elucidated, although their involvement has been confirmed in normal feeding, as well as in hyperalimentation, in both humans and several animal species [2]. These neuropeptides have been widely demonstrated to play an important role in the regulation of food intake, feeding, and energy metabolism [33,116]. By promoting food consumption, orexins are involved in feeding behaviors. In particular, several studies have shown that orexin neurons are activated by food deprivation and low blood glucose levels; in turn, orexin’s activation contributes to the stimulation of both hunger and food-seeking behaviors. On the other hand, the orexin system regulates both food preferences and anticipation of food rewards, making orexin a likely target for modulating reward-driven eating behavior. Recent data suggest that orexin signaling mediates reward-driven eating behavior and, within specific target regions, may regulate cue-induced overconsumption of palatable foods [117]. Furthermore, in high-fat diet (HFD)-fed mice, the orexin-dependent neural circuit of the paraventricular nucleus–lateral hypothalamus has been shown to be involved in reducing appetite and weight gain by improving insulin sensitivity and ultimately exerting a hypoglycemic effect [118]. In contrast, it has been observed that in wild-type mice fed a high-fat diet, low concentrations of bromocriptine can prevent obesity-induced glucose intolerance through peripheral, but not central, orexin-mediated mechanisms [119]. Another study demonstrated that melanin-concentrating hormone attenuates orexin-A-induced hedonic feeding in the ventral tegmental area of male mice fed a high-fat diet [120]. In rats exposed to prenatal stress, reduced plasma orexin-A concentrations are associated with anorexia nervosa and contribute to increased suppression of food intake and increased body weight loss [121]. Following sleep deprivation, the orexin system is strongly activated in both humans and animals [122,123], although it is still unclear to what extent its activation contributes to the increase in food reward seeking. Recently, it has been shown that in females, OXR signaling is preferentially recruited after deep sleep, which in turn preferentially activates OX_2_R signaling, increasing sucrose-based reward seeking. Furthermore, deep sleep disrupts coordinated activity across brain regions during reward seeking, an effect that in females is partially attenuated by OX_2_R antagonism [124].

Therefore, when promoting homeostatic food intake, orexin appears to act predominantly on OX_1_R in the dorsal and lateral regions of hypothalamus, where it may be responding to physiological signals such as a drop in glucose levels that occurs when animals most need to seek out and consume food [2]. Differently from homeostatic food intake, regarding palatable foods rich in fat or sugar, orexins promote their intake and significantly enhance their intake triggering a positive feedback loop between orexin and palatable foods. Additionally, a stronger orexin production and signal occur after each food intake episode, contributing to the overconsumption of fat and sugar [2].

Although the mechanisms by which orexin mediates its effects are still unclear, it is likely that orexin can mitigate several obesity-related dysfunctions; orexin-A, indeed, reduces weight gain in obesity-prone mice, while selective blockade of the OX_1_R reduces binge eating in rats [125]. Its multifaceted biological mechanisms have also established orexin as a possible neurobiological link between metabolic disorders and brain-related conditions [126]. Altogether, these observations suggest that orexin may be useful to better understand and discover biomarkers of metabolic vulnerability representing a novel target for the treatment of diet-related metabolic disorders, such as obesity/anorexia.

### Orexin in Metabolic Diseases: Obese vs. Normal Weight Subjects

**Figure 3:**
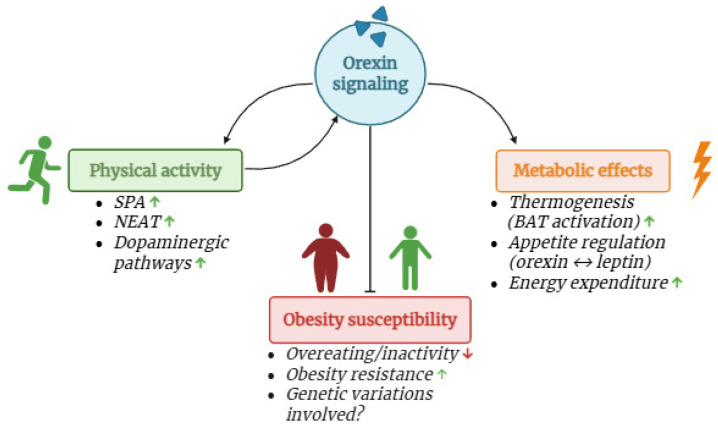
The complex relationship between orexin, physical activity and metabolic regulation. Image created in BioRender.com. Arrows indicate the direction of the signals.

Obesity continues to represent a worldwide health problem, currently affecting 30% of the population [127]. Obesity resistance is linked to SPA and varies among individuals [128]. Since orexin and SPA are deeply interconnected, it is important to analyze how this connection also regards obesity and obesity resistance (Figure 3). With fewer chemical signals to motivate responses, deficiencies in orexins are linked to physical inactivity and obesity. Animal research has shown that mice who lose their orexin-producing neurons are less physically active, have decreased energy metabolism, and are more likely to develop obesity and diabetes, even when they consume fewer calories [129,130,131].

Over the years, studies consistently reported lower orexin-A levels in obese individuals compared to their normal-weight counterparts. Baranowska et al. (2005) found that plasma orexin-A concentrations were markedly lower in obese women, particularly those with severe obesity (BMI > 40 kg/m^2^) [132]. This suggests that orexin-A plays a role in regulating body weight and energy balance. Numerous animal studies highlighted the beneficial effects of orexin in the resistance to obesity [70,133,134]. The diet-induced obesity (DIO) rat, i.e., the rat model for obesity, and the diet-resistant (DR) rat strand, have been often used to investigate the relationship between orexin and obesity. DR rats showed higher sensitivity to orexin injections, with a greater increase in NEAT compared to DIO rats [70].

The molecular mechanisms that are at the basis of the regulation of orexin in metabolic disorders are multiple. Beyond its involvement in feeding behavior, orexin is crucial in maintaining energy homeostasis [135]. It orchestrates energy expenditure, thermogenesis, and lipid metabolism through interactions with various metabolic centers in the brain. Orexin-producing neurons in the lateral hypothalamus are activated by both fasting and food deprivation, suggesting a role in promoting food-seeking behavior [136]. Activation of orexin neurons stimulates food intake, while their inhibition reduces food consumption [137]. On the other hand, orexin receptors are expressed in various tissues, including brown adipose tissue (BAT) and skeletal muscles, which are involved in thermogenesis and energy dissipation [138,139,140].

Its complex interactions with other hormones, such as leptin, further highlight its importance in maintaining energy balance [141]. Dysregulation of orexin signaling has been implicated in metabolic disorders, making it a potential pharmacological target for therapeutic interventions aimed at addressing obesity and related conditions [142].

Regarding metabolic conditions and obesity, we need to consider again that the relationship between orexin and PA is bidirectional, since one regulates the other. The observed elevation in plasma orexin-A levels following exercise indicates a positive feedback loop where PA boosts orexin levels, which in turn promotes further activity. This suggests that promoting PA could be a strategy to increase orexin levels and thereby enhance energy expenditure and weight loss in obese individuals. The higher orexin levels promoted by regular PA enhance alertness, motivation, and energy expenditure [143]. Indeed, orexin has been shown to activate mesolimbic dopamine pathways, which are critical in promoting motivation for obtaining rewards [144]. Specifically, orexin’s interaction with the ventral tegmental area (VTA) and the nucleus accumbens, key components of the reward circuitry, is crucial for effort-based tasks and decision-making [143].

Given the impact of orexin on NEAT and the complex interplay between energy expenditure and PA, it is important to explore additional mechanisms that may contribute to the aforementioned effects. One key area of interest is the role of thermogenesis in BAT, which plays a crucial role in regulating energy expenditure and maintaining body temperature [145]. Recent research has highlighted that orexin not only influences general PA but also directly affects thermogenic processes in BAT [146,147,148]. The influence of orexin on cardiovascular functions and sympathetic neurotransmission (e.g., increase of sympathetic firing in BAT) in animal models is documented, highlighting the broad functionality of this neuropeptide [148,149,150,151]. By enhancing BAT thermogenesis, orexin could significantly impact overall energy balance and contribute to variations in NEAT. Therefore, further investigating the relationship between orexin and BAT thermogenesis could provide valuable insights into the broader regulatory mechanisms governing energy expenditure and PA.

On the other hand, other research teams have uncovered how variations in genes encoding orexin and its receptors can influence individual differences in SPA and susceptibility to obesity. For instance, the pattern of orexin’s receptors expression was found to differ between two groups of rats undergoing a high-fat diet that were resistant or prone to diet-induced obesity, respectively [152]. Future research is required in order to elucidate the several genetic and circulating factors that underpin the biological processes of orexin.

Over time, numerous studies have concentrated on the correlation between orexin and PA. Animal studies have shown that orexin knockout mice exhibit reduced PA [153] and increased susceptibility to obesity, highlighting orexin’s role in energy balance. Accordingly, on the other hand, the administration of orexin in different brain areas enhances SPA [73,154,155]. In an analogue way, plasma orexin-A levels were positively correlated with PA in obese individuals, suggesting that higher orexin levels might encourage more PA and contribute to weight management also in humans [132]. Moreover, recent findings indicate that orexin levels are elevated following an exercise regimen in human subjects [31,156].

## 6. Orexin Role in Sleep Control and Disorders

**Figure 4:**
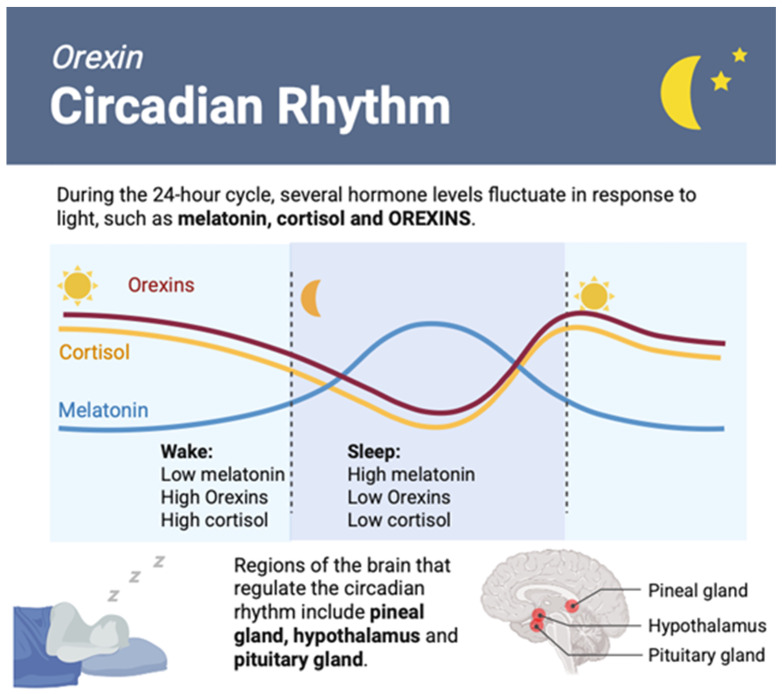
Orexin fluctuation in the circadian rhythm. Image created in BioRender.com.

It is hypothesized that a primary role of orexins is to control sleep and arousal, and the neurons that release orexins are most active during the day [142]. The neuropeptide stimulates other neurons to release neurotransmitters that promote alertness, such as dopamine, serotonin, and norepinephrine. Excessive orexin signaling can result in alterations of the sleep pattern, with reductions in the necessary hours of sleep at night. Sleep health is a fundamental component of the whole organism’s health, as it is related to several important physiological functions [157]; its disruption is linked to both short- and long-term consequences on wellbeing, starting from excessive daytime sleepiness and errors in judgement and arriving to increased risk of hypertension, diabetes, obesity, depression, heart attack, and stroke [158]. Sleep is a complex physiological process that consists of different stages, including rapid eye movement (REM) sleep. REM sleep is characterized by vivid dreaming, rapid eye movements, and muscle atonia. Alterations in the sleep cycle can result in deficits in the cognitive sphere, impacting functions such as attention, memory, and executive control [159]. Administration of orexin-A in mouse models increases wake and suppresses REM sleep [160]; while, during REM sleep, the activity of orexin maintains a state of wakefulness (Figure 4).

People diagnosed with type 1 narcolepsy have an 85% to 95% reduction in the number of neurons that produce orexins. This loss of orexin-producing neurons leads to the symptoms of narcolepsy, including excessive daytime sleepiness, sleep paralysis, hallucinations, and cataplexy. While weight gain is not a symptom of narcolepsy, people with this condition are more likely to be overweight [161]. Research suggests that the link between narcolepsy and weight gain may be related to orexin’s role in regulating physical activity.

However, when evaluating the cognitive deficits in conditions of sleep deprivation, it is important to consider that they can also be a direct consequence of lack of sleep per se, rather than of a dysfunction in the orexin system [67]. Since orexins stimulate wakefulness, blocking the effects of these neuropeptides might help to treat some sleep disorders. Dual orexin receptor antagonists (DORAs) are a new type of prescription sleep aid that targets the body’s orexin system. These medications work by acting as orexin receptor antagonists, meaning that they block the effects of orexins in the body, reduce the drive to stay awake, and facilitate sleep. Numerous polysomnographic studies have investigated the effects of orexin receptor antagonists on sleep architecture in both healthy individuals and patients with sleep disorders [162]. Orexin receptor antagonists improve sleep parameters and promote sleep continuity. Two types of DORAs are currently approved by the Food and Drug Administration (FDA) for the treatment of insomnia in adults: suvorexant and daridorexant. Newer DORAs are still in development.

Recently, new evidence has indicated that the orexinergic system is altered in several neurodegenerative diseases, such as Alzheimer’s disease, Parkinson’s disease, and multiple sclerosis, and its dysregulation plays a crucial role in the pathogenesis of these diseases [100,163,164,165,166].

Sleep disturbances are common in patients affected by Alzheimers, and related to neuroinflammation, orexin, and AD biomarkers in both AD patients and mice. In particular, short sleep duration in AD is associated with significant cognitive impairment, neuroinflammation, increased orexin and Aβ deposition, and altered sleep architecture [163]. These alterations precede Aβ deposition in the hippocampus and cognitive impairment in the mouse model [163]. Regarding the orexin expression, the results are not entirely consistent: in fact, some have observed a significant reduction in orexin-A levels in AD patients [101], while others (Slats et al. and Liguori et al.) have found no differences in orexin-A levels in the CSF between AD patients and healthy controls [103,164]. Gabelle et al. instead reported an increase in orexin-A in AD patients [165].

Sleep disruption is a symptom found in Parkinson patients [166,167] who suffer from excessive daytime sleepiness and sleep attacks [166,168,169]. Regarding the dysregulation of the orexinergic system in PD, the number of studies is limited and inconclusive. Indeed, Drouot et al. reported that orexin-A levels were reduced in the ventricular cerebrospinal fluid compared to controls [170], while Yasui et al. reported no differences between orexin-A levels in cerebrospinal fluid from patients with PD compared to controls [171].

Sleep disturbances are a trigger for relapses in MS, indicating a potential role for the orexinergic pathway in MS [172]. Furthermore, an orexin-A reduction in CSF was observed in a MS female patient with hypersomnia [173]. However, no significant orexin-A deficiency in MS patients compared to controls was detected by Constantinescu et al. and Burfeind et al. [174,175]. Furthermore, Gencer et al. observed a significant reduction in orexin-A levels in MS patients; orexin-A levels related to MS progression [114].

## 7. Orexin in Clinical Trials

Orexin-A pharmacology has been investigated primarily through the use of selective orexin-1 receptor antagonists (SO1RAs), which have emerged as promising candidates for the treatment of substance use disorders, eating disorders, obsessive-compulsive disorder, and anxiety-related conditions [176]. In contrast, dual orexin receptor antagonists (DORAs), which block both OX_1_R and OX_2_R, are generally considered less suitable for these indications because of their pronounced sleep-promoting properties. Well-known examples include suvorexant (Belsomra), lemborexant (Dayvigo), and daridorexant (Quviviq), all of which are FDA-approved for the management of insomnia.

Beyond their established role in sleep regulation, however, these compounds are being actively studied for broader applications. For example, suvorexant has been evaluated in clinical studies exploring its efficacy in major depressive disorder and opioid use disorder, showing preliminary benefit in modulating sleep-related and affective symptoms. Lemborexant, a competitive antagonist with higher selectivity for OX_2_R, has also been tested in trials investigating its effects on circadian rhythm disorders and psychiatric comorbidities. In addition, seltorexant, a selective OX_2_R antagonist, has progressed to phase III clinical trials as an adjunctive treatment for depression. Other compounds, such as filorexant and daridorexant, remain in earlier stages of investigation, with phase II studies currently focusing on dose optimization, tolerability, and efficacy outcomes.

Conversely, orexin receptor agonists are being developed to enhance orexin signaling, offering a novel therapeutic strategy for central hypersomnia syndromes, including narcolepsy and idiopathic hypersomnia. Several pharmaceutical companies, including Eisai and Centessa, are leading the development of compounds with high selectivity for OX2R. Among them, ORX750 has advanced to phase II clinical trials assessing safety, tolerability, and efficacy outcomes, while ORX142 is being tested in phase I studies in healthy volunteers to evaluate pharmacokinetics and safety. Eisai’s E2086 has also entered early-stage trials in narcoleptic patients, providing important proof-of-concept data for OX_2_R agonism. Perhaps most notably, TAK-861 has progressed to phase III evaluation, reflecting the growing recognition of orexin agonists as a potentially transformative class of drugs in sleep medicine.

## 8. Conclusions

The orexinergic system is known to be a key regulator of several pathophysiological mechanisms, including energy metabolism and physical activity, with significant implications for obesity, aging, and neurodegenerative diseases. Despite significant progress, many questions remain about the precise mechanisms by which orexin influences physical activity, energy balance, and the sleep–wake cycle. Indeed, although substantial evidence exists linking orexin to maintaining motivation and the ability to engage in regular physical activity, and thus to improved metabolic and brain health, the underlying molecular relationship linking orexin to metabolic and neurodegenerative diseases remains complex and requires further investigation. For these reasons, modulating orexin levels represents a potential therapeutic target for the treatment of most metabolic disorders. Furthermore, understanding the role of orexin in sleep disturbances and fatigue could lead to new treatments aimed at alleviating the symptoms of neurodegenerative diseases. Orexin and its receptor system is a high-value therapeutic target. The field has already produced marketed drugs (insomnia treatments) and late-stage clinical candidates (for narcolepsy), with growing evidence of broader applications in psychiatry and neurology. Future research should focus on longitudinal studies and advanced neuroimaging techniques to provide insights into the role of orexin in health and disease, paving the way for targeted interventions. Finally, the growing interest in orexin-based drugs for various neurological and psychiatric disorders calls for further research into their hormonal effects, as well as alterations in their pharmacology during periods of gonadal hormonal fluctuations.

## 9. Methods

In this review, we analyzed published articles from the most recent literature, providing a balanced and comprehensive overview of the most important discoveries in relation to pathogenesis and possible biomarkers and target molecules of orexin. The databases used were PubMed, Scopus, Web of Science, and Google Scholar, using appropriate keywords (orexin; physical activity; sleep; nutrition; neurodegeneration). Out of approximately 450 papers (original articles, systematic, and meta-analysis reviews), 176 were chosen and considered appropriate for this focused review. Other papers related to the chosen keywords were discarded, as they were considered to be outside the scope of the research. The time period chosen for the literature search was from 1998 to date. The bibliographic research has been divided into five different steps and has followed inductive reasoning.

In the first step, the research was focused on papers regarding “orexin”; in the second step, the “orexin and physical activity”; in the third step, the “sleep and orexin”; in the fourth step, the “nutrition and orexin” and in the fifth step, the “orexin and neurodegeneration”.

## Abbreviations

The following abbreviations are used in this manuscript:

OXR	Orexin receptor
HPA	Hypothalamic–pituitary–adrenal
PA	Physical activity
SPA	Spontaneous physical activity
NEAT	Non-exercise activity thermogenesis
BDNF	Brain-derived neurotrophic factor
IGF-1	Insulin-like growth factor 1
PLC	Phospholipase C
PLD	Phospholipase D
MAPK	Mitogen-activated protein kinase
AD	Alzheimer’s disease
PD	Parkinson’s disease
CSF	Cerebrospinal fluid
PVN	Paraventricular nucleus
MS	Multiple sclerosis
Aβ	Amyloid-β
HFD	High-fat diet
DIO	Diet-induced obesity
DR	Diet-resistant
VTA	Ventral tegmental area
BAT	Brown adipose tissue
REM	Rapid eye movement
DORAs	Dual orexin receptor antagonists
FDA	Food and Drug Administration
SO1RAs	Selective orexin-1 receptor antagonists
